# Chronic Renal Failure, Cachexia, and Ghrelin

**DOI:** 10.1155/2010/648045

**Published:** 2010-02-04

**Authors:** A. Laviano, Z. Krznaric, K. Sanchez-Lara, I. Preziosa, A. Cascino, F. Rossi Fanelli

**Affiliations:** ^1^Department of Clinical Medicine, Sapienza University of Rome, 00185 Rome, Italy; ^2^Department of Internal Medicine, Rebro Division of Gastroenterology, University Hospital Center, 10 000 Zagreb, Croatia; ^3^Oncology Center Diana Laura Riojas de Colosio, Médica Sur Clinic and Foundation, 14050 México City, Mexico

## Abstract

Protein energy wasting is frequently observed in patients with advanced chronic renal failure and end-stage renal disease. Anorexia and reduced food intake are critical contributing factors and negatively impact on patients' survival. Ghrelin is a prophagic peptide produced by the stomach and acting at the hypothalamic level to increase the activity of orexigenic neurons. In patients with chronic renal disease, plasma levels are increased as a likely effect of reduced renal clearance. Nevertheless, patients' food intake is significantly reduced, suggesting inflammation-mediated resistance of hypothalamic nuclei to peripheral signals. A number of forms of evidence show that ghrelin resistance could be overcome by the administration of exogenous ghrelin. Therefore, ghrelin has been proposed as a potential strategy to improve food intake in chronic renal failure patients with protein energy wasting. Preliminary data are encouraging although larger prospective clinical trials are needed to confirm the results and to identify those patients who are likely to benefit most from the administration of exogenous ghrelin.

## 1. Introduction

The clinical course of chronic diseases is frequently complicated by the progressive deterioration of nutritional status, which significantly impacts on patients' morbidity, mortality and quality of life [1]. Chronic renal failure and end-stage renal disease are not exceptions, and up to 60% of patients undergoing haemodialysis present with malnutrition [2]. It is important to remember that malnutrition in end-stage renal disease is a complex syndrome, which develops not only from reduced energy intake like in simple starvation. Indeed, profound metabolic changes (i.e., increased protein catabolism, reduced muscle anabolism, increased energy expenditure, adipose tissue loss, insulin resistance, etc.) are critical determining factors. Consequently, normalization of energy and protein intake, although it may improve renal patients' clinical outcome [3], does not result in restoration of nutritional status and body composition. 

 Although the clinical phenotype of malnourished patients with chronic renal failure can be easily recognized and is widely accepted, more uncertainties exist on the terminology to be used to define this syndrome. Indeed, the word “malnutrition” may be misleading since it may generate confusion with the deterioration of nutritional status induced by simple starvation only. The term “cachexia” has been largely used to define disease-associated malnutrition and has been proposed to include malnourished renal patients as well [4]. However, considering the clinical specificities of end-stage renal disease, a number of different terms have been also suggested, including “protein-energy wasting” [5], “malnutrition-inflammation complex (or cachexia) syndrome” [2], and “kidney disease wasting” [6]. For the purpose of this review article and in the attempt to highlight the analogies with other chronic conditions, the term “cachexia” will be used to define the clinical syndrome of weight loss, anorexia, reduced muscle performance, anemia, and so forth, associated with chronic renal failure and end-stage renal disease. 

 The pathogenesis of cachexia in renal patients is multifactorial, but anorexia and reduced food intake, as well as profound changes in macronutrient metabolism are the driving forces, leading to a practically not reversible catabolic status. The molecular mechanisms prompting these clinical symptoms are increasingly being understood. As recently reviewed by Muscaritoli et al., increased levels of circulating cytokines, metabolic acidosis, oxidative stress and insulin resistance all appear to be variably implicated, although the individual role of each component in the pathogenesis of chronic kidney disease-related cachexia is still unclear [5]. However, it appears from recent clinical data that inflammation may play a pre-eminent role in triggering the cascade of biochemical events eventually leading to the development of anorexia and muscle wasting, that is, to cachexia.

## 2. Chronic Renal Failure and Cachexia

As previously mentioned, one of the most important player in the pathogenesis of cachexia in renal patients is the reduction of appetite, that is, anorexia. In a large study involving a cohort of 331 patients undergoing maintenance hemodialysis, the presence of impaired appetite (reported by 38% of the patients studied) was significantly associated with reduced 12-month survival and increased hospitalization rate [7]. Interestingly, anorexia was associated with increased circulating levels of surrogate markers of inflammation, that is, Tumor Necrosis Factor-*α* and C-reactive protein [7]. These data points to inflammation as the major trigger of the molecular cascade of events eventually leading to anorexia and poor outcome. 

 Supportive data have been published and indicate that inflammation may also trigger the progressive wasting of skeletal muscles. A number of experimental and clinical studies consistently show that muscle mass wasting is closely related to the presence of inflammation and in particular to inflammation-mediated activation of specific proteases [5, 8, 9]. In uremic rodents and patients, the first step in muscle protein loss is the activation of caspase-3. This cleaves the complex structure of muscle, thereby exposing a characteristic 14 kDa actin fragment in the insoluble fraction of muscle. Then, the ubiquitin-proteasome system is activated, which rapidly degrades proteins released by caspase-3 cleavage of muscle proteins. 

 In adults and under physiological conditions, muscle protein catabolism and anabolism are in equilibrium and offset each other. During disease, including end-stage renal failure, the activation of the proteolytic pathways is not counterbalanced by a corresponding increase of the anabolic pathways [9]. Indeed, activation of the proteolytic systems occurs when there is suppression of the growth-hormone-(GH-) mediated cellular signaling pathway activated by the insulin/insulin-like growth factor (IGF) 1, the phosphatidylinositol 3-kinase/Akt pathway, the main muscle protein synthesis pathway [9]. It is important to note that renal failure is a state of GH resistance and not GH deficiency [10]. Some mechanisms of GH resistance are: reduced density of GH receptors in target organs, impaired GH-activated post-receptor Janus kinase/signal transducer and activator of transcription (JAK/STAT) signaling, and reduced levels of free IGF-1 due to increased inhibitory IGF-binding proteins [11]. 

 End-stage renal disease is associated with a state of insulin resistance [12]. Convincing data show that insulin resistance is associated with increased skeletal muscle protein breakdown [13], and that inflammation represents a determining factor [14]. Oxidative stress could also influence insulin sensitivity of peripheral tissues [14]. Interestingly, inflammation has been demonstrated to derange mitochondrial function thereby favoring leakage of reactive oxygen species [15]. Since mitochondrial dysfunction contributes to the development of insulin resistance in skeletal muscle [16], it appears that inflammation either directly, that is, activating the proteolytic systems, or indirectly, that is, favoring the development of oxidative stress and insulin resistance, is the main mediator of wasting in patients with chronic renal failure. Also, considering its role in anorexia [17], it could be speculated that disease-induced inflammation triggers the cascade of biochemical events leading to cachexia, although the specific phenotype of cachexia of each renal patients is also determined by his/her own genetic profile [17]. 

 Supporting the role of the central nervous system as a preferential target for inflammation in mediating the onset of cachexia in renal patients [17], consistent evidence show that hypothalamic melanocortin signaling triggers anorexia and skeletal muscle wasting in experimental models of uremic cachexia [18], and thus provide a further potential target for the development of effective therapies [19].

## 3. Ghrelin in the Pathogenesis of Cachexia of Renal Patients

Under physiological conditions, energy homeostasis is tightly controlled by the hypothalamic integration of peripheral signals conveying to the central nervous system information on the metabolic status of peripheral tissues [20]. In the hypothalamus, two populations of neurons are colocalized in the rodents' arcuate nucleus (the infundibular nucleus in humans): the activation of pro-opiomelancortin (POMC) neurons promotes increased energy expenditure and satiety, while activation of Neuropeptide Y (NPY) neurons triggers the onset of appetite [21]. The integrated activities of POMC and NPY neurons are controlled by a complex mechanism: basically, POMC and NPY neurons reciprocally respond to peptides, as well as other signals, produced in peripheral tissues according to specific metabolic conditions, and modulate accordingly energy homeostasis [21]. 

 Ghrelin is a unique hormone with potent orexigenic effects [22]. It is an acylated peptide produced primarily by gastric cells representing the endogenous ligand for the growth hormone secretagogue receptor [23]. In addition to stimulate the release of GH from the pituitary, ghrelin administration stimulates food intake, and carbohydrate utilization, and increases adiposity in rodents, suggesting a role for this hormone in energy balance [24]. Ghrelin influences neuronal activity through its receptor in several areas of the brain governing energy homeostasis, including the hypothalamus (specifically arcuate NPY neurons) [24]. Additionally, orexigenic effects of ghrelin are also mediated by modulation of hypothalamic fatty acid metabolism [25]. 

 Under physiological conditions, acylated ghrelin, that is, the orexigenic form of ghrelin, represents <10% of circulating ghrelin, the rest being des-acylated ghrelin. Des-acylated ghrelin has been considered as the inactive form of ghrelin, but recent data suggest that it may exert biological functions [26]. 

 Considering its contributory role in determining energy homeostasis, ghrelin has been postulated to be involved in the pathogenesis of renal cachexia. In particular, reduced levels of circulating ghrelin were hypothesized as a pathogenetic mechanism mediating anorexia. Experimental and clinical studies could not support this hypothesis, since ghrelin levels have been consistently found increased in patients with end-stage renal failure, undergoing or not maintenance hemodialysis [27–29]. Supporting the lack of a major role for acylated ghrelin in mediating uremic anorexia, Bossola et al. recently demonstrated that circulating levels are significantly higher in uremic patients with poor/very poor appetite when compared with uremic patients with good/fair appetite [30]. More recently, Zabel et al. showed that in hemodialysis patients, hunger ratings measured with visual analogue scales correlate with markers of inflammation, but no correlation can be found with circulating acylated ghrelin [31]. On the other hand, des-acylated ghrelin has been postulated to suppress food intake, and Muscaritoli et al. demonstrated higher levels of des-acylated ghrelin in anorexic uremic patients undergoing hemodialysis than in non-anorexic patients [32]. However, the role of des-acylated ghrelin in mediating uremic anorexia needs to be further investigated. 

 The acylation of ghrelin is mediated by a specific enzyme, ghrelin O-Acyltransferase (GOAT), which attaches octanoate to serine-3 of ghrelin. Considering the potential pathogenic role of the ratio in plasma between acylated and des-acylated ghrelin in mediating renal cachexia [33], it would be important to measure the expression and activity of GOAT during chronic renal failure. Unfortunately, such data are not available yet. 

 The mechanisms responsible for the increase of circulating ghrelin levels during end-stage renal failure are being investigated, and include impaired clearance and/or metabolism of ghrelin in the kidney. In contrast, the etiology of renal failure and hemodynamic parameters do not appear have any effect on plasma ghrelin levels [34]. Quite recently, the concept that ghrelin is a passive bystander influenced by progressive renal failure has been challenged by a series of intriguing results. Wang et al. demonstrated that ghrelin protects against endotoxemia-induced acute kidney injury by a likely inhibition of proinflammatory cytokines [35]. Barazzoni et al. showed in nondiabetic maintenance hemodialysis patients that insulin sensitivity is associated negatively with systemic inflammation and positively with total plasma ghrelin, suggesting a potential novel role of ghrelin in preserving insulin sensitivity in maintenance hemodialysis [36]. 

 The specific role of ghrelin in muscle wasting of renal patients with cachexia remains to be determined. Under physiological conditions, plasma ghrelin levels relate to muscle mass [37], likely because of its orexigenic and GH-releasing effects. More recently, Sheriff et al. showed that a ghrelin receptor agonist attenuates muscle wasting in a model of burn injury-induced proteolysis [38]. It could be postulated that renal cachexia is associated with ghrelin resistance, which in turn may limit the anticatabolic effects of ghrelin, thereby exacerbating muscle proteolysis. This hypothesis needs to be tested in experimental models and in clinical trials. 

 When considered together, these data indicate that during end-stage renal disease, increased circulating ghrelin levels may represent an attempt to compensate and counteract inflammation, and support the use of exogenous ghrelin as a potential therapy for renal cachexia.

## 4. Ghrelin in the Therapy of Cachexia of Renal Patients

As previously mentioned, renal failure is a state of GH resistance, and based on the available data, it is a state of ghrelin resistance too. However, as previously mentioned, increased ghrelin levels could be considered as a protective mechanism to counteract the detrimental metabolic effects induced by inflammation. In this regard, exogenous administration of the hormone may overcome ghrelin resistance at target organs, improve metabolic alterations and result in clinical benefit. 

 In a pilot study testing the effects of a single subcutaneous administration of ghrelin in mild/moderate malnourished uremic patients receiving peritoneal dialysis, energy intake immediately doubled and was not followed by subsequent underswing [39]. These positive preliminary results have been confirmed in a more recent 7-day trial, which confirmed that in malnourished dialysis patients, daily subcutaneous ghrelin administration immediately and significantly increased appetite, with an increase in energy intake noted at the first study meal [40]. More importantly, this effect persisted throughout the week without the occurrence of clinically relevant side effects [40]. Also, energy expenditure, measured with free-living pulse and motion monitors, was unchanged by ghrelin [40]. The mechanisms by which ghrelin administration ameliorate energy intake of uremic patients is currently being investigated. In an experimental model of chronic renal failure, ghrelin treatment resulted in increased food intake and an improvement in lean body mass accrual that was related in part to a decrease in muscle protein degradation [41]. Additionally, circulating inflammatory cytokines were reduced in nephrectomized animals by ghrelin treatment relative to saline treatment. Finally, ghrelin-treated animals showed a decrease in the expression of IL-1 receptor in the brainstem and a decrease in expression of prohormone convertase 2, an enzyme involved in the processing of proopiomelanocortin to the anorexigenic peptide *α*-MSH [41]. More recently, Barazzoni et al. found that ghrelin treatment normalized low muscle mitochondrial enzyme activities in uremic rats [42]. This effect was associated with a lower muscle triglyceride content and higher AKT phosphorylation. Interestingly, the effects of ghrelin on mitochondria are independent of changes in food intake, while combined ghrelin treatment and higher food intake were needed to enhance AKT phosphorylation [42]. When these effects are considered together, it appears that ghrelin-induced muscle mitochondrial changes and lower tissue triglycerides could favor insulin action and muscle anabolism in the presence of improvement in food intake. 

 The administration of ghrelin to patients with renal cachexia may yield clinical benefits beyond its anticatabolic effects. In particular, chronic renal failure is frequently associated with a number of cardiovascular alterations, which may be counteracted by exogenous ghrelin. As an example, preliminary data indicate that ghrelin restores endothelial dysfunction in patients with obesity-related metabolic syndrome [43].

## 5. Conclusion

Cachexia associated with chronic renal failure is a clinically relevant syndrome, which negatively impacts on patients' morbidity and mortality, and impinges on their quality of life. The pathogenesis of this syndrome is quite complex, but inflammation represents a preeminent feature triggering the cascade of biochemical events eventually leading to deterioration of nutritional status. Ghrelin is a unique orexigenic hormone, whose role in cachexia is to counteract the detrimental effects on targeted organs of inflammation and oxidative stress. Although a state of ghrelin resistance appears to develop during end-stage renal failure, exogenous administration of ghrelin appears a promising therapeutic strategy to improve the clinical outcome of uremic patients.
